# Supplementary figures and images for: Insights into the genetic diversity and population structure of prevalent Theileria orientalis in Bangladesh
Source: PLoS One. 2026 Feb 18;21(2):e0334382. doi: 10.1371/journal.pone.0334382 (PMC12915967; doi:10.1371/journal.pone.0334382)

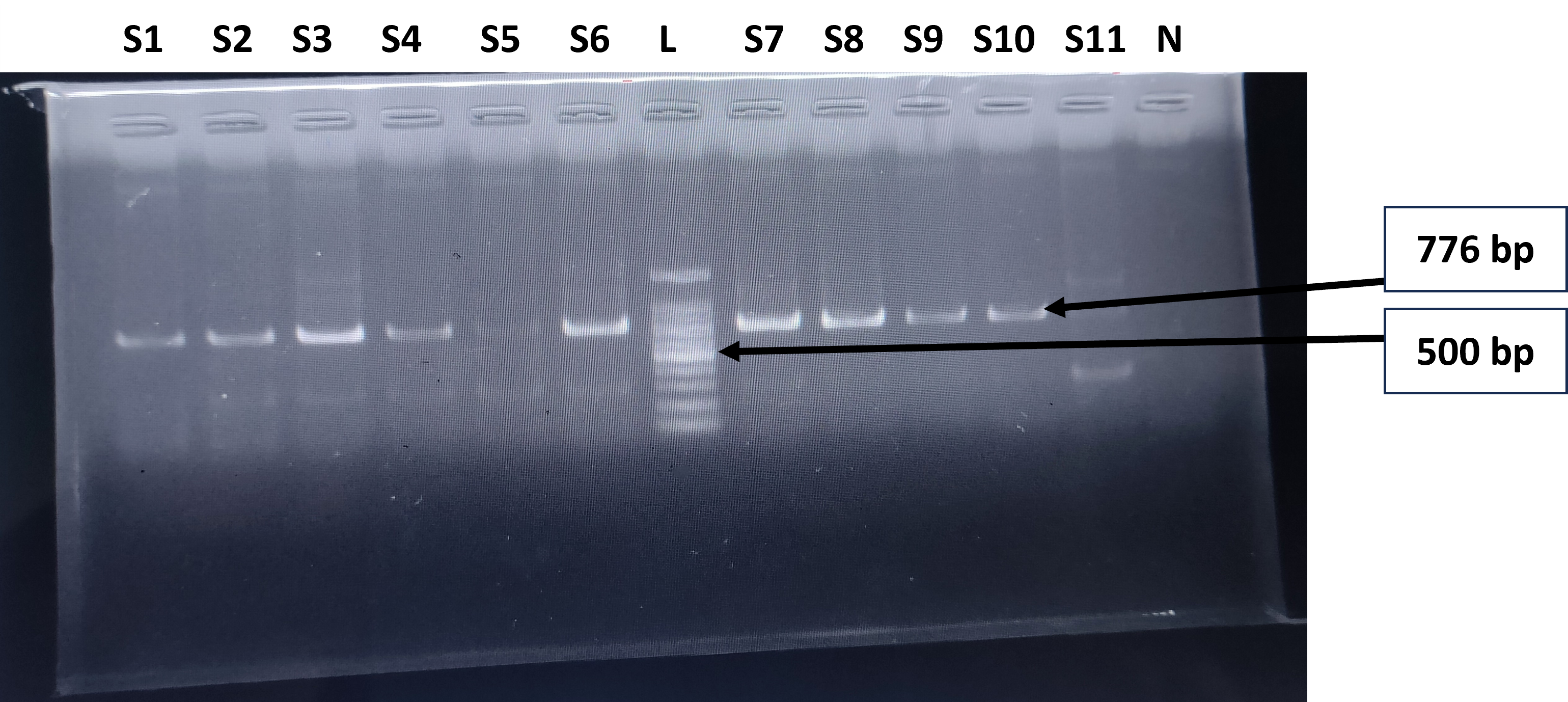

Supplement: S1 Fig — Lane L: 100 bp DNA ladder, Lane S1-S11: Test samples and Lane N: Negative control. All data and analysis scripts supporting this study are archived and available at https://doi.org/10.5281/zenodo.17191643 (TIF) [file pone.0334382.s001.tif]
