# Supplementary material for: Insights into the genetic diversity and population structure of prevalent Theileria orientalis in Bangladesh
Source: PLoS One. 2026 Feb 18;21(2):e0334382. doi: 10.1371/journal.pone.0334382 (PMC12915967; doi:10.1371/journal.pone.0334382)
Supplement: Attachment — Submitted filename: Response to Reviewers.docx [file pone.0334382.s003.docx]

Manuscript Title: Insights into the genetic diversity and population structure of prevalent *Theileria* *orientalis* in Bangladesh

**Manuscript Number:** PONE-D-25-52299

Journal: PLOS ONE

Dear Editor

We sincerely appreciate the time and effort you have taken to review our manuscript. Your insightful and constructive comments have greatly helped us to improve the quality and clarity of the paper. We have carefully revised the manuscript in accordance with your suggestions.

Below, we provide a detailed, point-by-point response to each comment.

- Reviewer comments are presented in bold.
- Our responses follow beneath each comment in regular text.
- The revised manuscript clearly indicates all changes, highlighted in light blue, and corresponding responses are provided in this rebuttal letter.

We hope that our revisions satisfactorily address all concerns and that the manuscript is now suitable for consideration in *PLOS ONE*.

With Kindest regards

(Professor Dr. MD H. Talukder)

on behalf of all authors

**Authors’ responses to the comments related to the journal requirements**

**1. Please ensure that your manuscript meets PLOS ONE's style requirements, including those for file naming. The PLOS ONE style templates can be found at**

[**https://journals.plos.org/plosone/s/file?id=wjVg/PLOSOne_formatting_sample_main_body.pdf**](https://journals.plos.org/plosone/s/file?id=wjVg/PLOSOne_formatting_sample_main_body.pdf) **and**

[**https://journals.plos.org/plosone/s/file?id=ba62/PLOSOne_formatting_sample_title_authors_affiliations.pdf**](https://track.editorialmanager.com/CL0/https:%2F%2Fjournals.plos.org%2Fplosone%2Fs%2Ffile%3Fid=ba62%2FPLOSOne_formatting_sample_title_authors_affiliations.pdf/1/010f019abf3601f9-11d62d82-7d54-4723-be46-3b93b8f430b8-000000/uB_eUWDsigpZhYamw2vMGxuCeXf0V2UfoHGGB00-V4A=239)

**Response:**

Thank you for the reviewer’s suggestion. We have carefully revised the manuscript to ensure that it fully meets the PLOS ONE style requirements. All formatting has been updated according to the PLOS ONE templates for the main body and for the title/authors/affiliation’s sections. Additionally, all files have been renamed following the journal’s file-naming guidelines.

**2. Thank you for stating the following financial disclosure:**

**“The authors acknowledge the financial support (Principal investigator, M. H. T.; research and innovation subproject; project code: (RP-C-03-24) from the Livestock and Dairy Development Project (LDDP), jointly funded by the World Bank and Department of Livestock Services (DLS), Ministry of Fisheries and Livestock, Govt. of Bangladesh.”**

**Please state what role the funders took in the study.  If the funders had no role, please state: "The funders had no role in study design, data collection and analysis, decision to publish, or preparation of the manuscript."**

**If this statement is not correct you must amend it as needed.**

**Please include this amended Role of Funder statement in your cover letter; we will change the online submission form on your behalf.**

**Response:**

We confirm that the funders’ contribution was limited solely to financial support. They were not engaged in designing the study, data acquisition, data analysis, manuscript drafting, or publication decisions. Accordingly, the required statement has been added to the cover letter.

**3. Please note that your Data Availability Statement is currently missing the repository name. If your manuscript is accepted for publication, you will be asked to provide these details on a very short timeline. We therefore suggest that you provide this information now, though we will not hold up the peer review process if you are unable.**

**Response:**

The required data repository details have been provided in the cover letter. Please note that the link is now included as recommended. All data and analysis scripts supporting this study are archived and available at https://doi.org/10.5281/zenodo.17191643

**4. Please include your full ethics statement in the ‘Methods’ section of your manuscript file. In your statement, please include the full name of the IRB or ethics committee who approved or waived your study, as well as whether or not you obtained informed written or verbal consent. If consent was waived for your study, please include this information in your statement as well.**

**Response:**

We appreciate the reviewer’s observation. The full ethics statement has been added to the methodology section. Specifically, we have provided the complete name of the Institutional Ethics Committee that approved the study and clarified the consent procedure.

**5. We note that Figure 1 in your submission contain map images which may be copyrighted. All PLOS content is published under the Creative Commons Attribution License (CC BY 4.0), which means that the manuscript, images, and Supporting Information files will be freely available online, and any third party is permitted to access, download, copy, distribute, and use these materials in any way, even commercially, with proper attribution. For these reasons, we cannot publish previously copyrighted maps or satellite images created using proprietary data, such as Google software (Google Maps, Street View, and Earth). For more information, see our copyright guidelines:** [**http://journals.plos.org/plosone/s/licenses-and-copyright**](http://journals.plos.org/plosone/s/licenses-and-copyright)**.**

**We require you to either (1) present written permission from the copyright holder to publish these figures specifically under the CC BY 4.0 license, or (2) remove the figures from your submission:**

**1. You may seek permission from the original copyright holder of Figure 1 to publish the content specifically under the CC BY 4.0 license.**

**We recommend that you contact the original copyright holder with the Content Permission Form (**[**http://journals.plos.org/plosone/s/file?id=7c09/content-permission-form.pdf**](http://journals.plos.org/plosone/s/file?id=7c09/content-permission-form.pdf)**) and the following text:**

**“I request permission for the open-access journal PLOS ONE to publish XXX under the Creative Commons Attribution License (CCAL) CC BY 4.0 (**[**http://creativecommons.org/licenses/by/4.0/**](http://creativecommons.org/licenses/by/4.0/)**). Please be aware that this license allows unrestricted use and distribution, even commercially, by third parties. Please reply and provide explicit written permission to publish XXX under a CC BY license and complete the attached form.”**

**Please upload the completed Content Permission Form or other proof of granted permissions as an "Other" file with your submission.**

**In the figure caption of the copyrighted figure, please include the following text: “Reprinted from [ref] under a CC BY license, with permission from [name of publisher], original copyright [original copyright year].”**

**2. If you are unable to obtain permission from the original copyright holder to publish these figures under the CC BY 4.0 license or if the copyright holder’s requirements are incompatible with the CC BY 4.0 license, please either i) remove the figure or ii) supply a replacement figure that complies with the CC BY 4.0 license. Please check copyright information on all replacement figures and update the figure caption with source information. If applicable, please specify in the figure caption text when a figure is similar but not identical to the original image and is therefore for illustrative purposes only.**

**The following resources for replacing copyrighted map figures may be helpful:**

**USGS National Map Viewer (public domain):** [**http://viewer.nationalmap.gov/viewer/**](http://viewer.nationalmap.gov/viewer/)

**The Gateway to Astronaut Photography of Earth (public domain):** [**http://eol.jsc.nasa.gov/sseop/clickmap/**](http://eol.jsc.nasa.gov/sseop/clickmap/)

**Maps at the CIA (public domain):** [**https://www.cia.gov/library/publications/the-world-factbook/index.html**](https://www.cia.gov/library/publications/the-world-factbook/index.html) **and** [**https://www.cia.gov/library/publications/cia-maps-publications/index.html**](https://www.cia.gov/library/publications/cia-maps-publications/index.html)

**NASA Earth Observatory (public domain):** [**http://earthobservatory.nasa.gov/**](http://earthobservatory.nasa.gov/)

**Landsat:** [**http://landsat.visibleearth.nasa.gov/**](http://landsat.visibleearth.nasa.gov/)

**USGS EROS (Earth Resources Observatory and Science (EROS) Center) (public domain):** [**http://eros.usgs.gov/#**](http://eros.usgs.gov/)

**Natural Earth (public domain):** [**http://www.naturalearthdata.com/**](http://track.editorialmanager.com/CL0/http:%2F%2Fwww.naturalearthdata.com%2F/1/010f019abf3601f9-11d62d82-7d54-4723-be46-3b93b8f430b8-000000/VTQZqIdUZSP5GO2wMvfmGTXXMPHtI2gTcW5bzmCiRKs=239)

**Response:**

We appreciate the editor’s guidance regarding copyright. The previously submitted map has now been replaced with a newly generated map of the study area, created entirely by the authors. No copyrighted material was used in this new figure and therefore no permission is required. The figure caption has been updated accordingly.

**6. PLOS ONE now requires that authors provide the original uncropped and unadjusted images underlying all blot or gel results reported in a submission’s figures or supporting information files. This policy and the journal’s other requirements for blot/gel reporting and figure preparation are described in detail at** [**https://journals.plos.org/plosone/s/figures#loc-blot-and-gel-reporting-requirements**](https://journals.plos.org/plosone/s/figures#loc-blot-and-gel-reporting-requirements) **and** [**https://journals.plos.org/plosone/s/figures#loc-preparing-figures-from-image-files**](https://journals.plos.org/plosone/s/figures#loc-preparing-figures-from-image-files)**. When you submit your revised manuscript, please ensure that your figures adhere fully to these guidelines and provide the original underlying images for all blot or gel data reported in your submission. See the following link for instructions on providing the original image**

**data:** [**https://journals.plos.org/plosone/s/figures#loc-original-images-for-blots-and-gels**](https://journals.plos.org/plosone/s/figures#loc-original-images-for-blots-and-gels)**.**

**In your cover letter, please note whether your blot/gel image data are in Supporting Information or posted at a public data repository, provide the repository URL if relevant, and provide specific details as to which raw blot/gel images, if any, are not available. Email us at** [**plosone@plos.org**](mailto:plosone@plos.org) **if you have any questions.**

**Response:**

We thank the editors for this requirement. The previously included gel image has been replaced because it was cropped. We have now provided a new version of the gel image that is original, uncropped, and unadjusted, in full compliance with PLOS ONE blot/gel reporting guidelines. The corresponding original underlying image has been included in the Supporting Information.

If additional gel or blot images or further raw data are required, we would be happy to provide them.

**8. Please review your reference list to ensure that it is complete and correct. If you have cited papers that have been retracted, please include the rationale for doing so in the manuscript text, or remove these references and replace them with relevant current references. Any changes to the reference list should be mentioned in the rebuttal letter that accompanies your revised manuscript. If you need to cite a retracted article, indicate the article’s retracted status in the References list and also include a citation and full reference for the retraction notice.**

**Response:**

We thank the reviewer for this important reminder. We have carefully reviewed the reference list to ensure that all citations are complete, accurate, and up to date. Any references that have been retracted have been either removed or replaced with relevant current literature. In cases where it was necessary to cite a retracted article, we have clearly indicated its retracted status in the reference list and included a citation to the retraction notice in the manuscript text. All changes to the reference list have been highlighted in the tracked changes and are noted in this rebuttal letter.

**Additional Editorial Comments:**

#reviewer 1:

- **Lines 35-36: Please change the sentence to: *“In contrast, T. orientalis does induce lymphoproliferation.”***

**Response:**

We have revised the sentence as suggested. The revised text now reads: In contrast, *T. orientalis* does induce lymphoproliferation. The manuscript has been revised to include this information in the introduction section (**Line 36-37**).

- **Lines 36-37: Please provide a reference for the tick vectors. I do not believe all tick species listed act as vectors.**

**Response**

We thank the reviewer for pointing out this. We have carefully reviewed the literature and updated the tick vector information to include only species confirmed as vectors of Theileria orientalis. The manuscript has been revised to include this information in the introduction section (**Line 37**). Appropriate references have also been added.

- **Please remove the abbreviation “Md.” from the author names.**

**Response**

We thank the reviewer for the suggestion. “Md” is an integral part of the authors’ official names. Following the journal’s suggestion, we have removed the dot after “Md.”

**Please delete the Acknowledgments section, as it is not relevant.**

**Response**

Thank you for the comment. The Acknowledgments section has been removed from the revised manuscript as requested.

- **Please ensure that the bibliography follows PLOS ONE formatting guidelines.**

**Response**

We appreciate the reviewer’s observation. The entire bibliography has been checked and reformatted according to the PLOS ONE reference style, including authorship format, article titles, journal names, volume/issue numbers, page ranges, and DOI formatting.

- **Since the study focuses on *T. orientalis*, which causes a disease distinct from other *Theileria* species, please be specific throughout the manuscript. Avoid using the general term “theileriosis” for *T. orientalis* infection (e.g., line 236).**

**Response**

Thank you for this important clarification. We agree that Theileria orientalis infection is clinically and epidemiologically distinct from bovine theileriosis caused by T. annulata or T. parva. In the revised manuscript, we have replaced all general uses of the term “theileriosis” with the specific phrase “T. orientalis **(line 16, 18, 22, 25, 36, 37, 39, 42, 47, 61, 63, 67, 69, 75, 77, 78, 111, 115, 121, 130, 135, 141, 154, 155-158, 161,174,185, 188,200, 206, 216, 223, 243, 257-259, 263, 267, 270, 279, 281, 287, 289, 296, 303, 305, 307, 310, 314, 316, 321, 328, 353, 355, 358, 362-363, 366-367)** infection” or oriental theileriosis (**line 45,46,60,75,81,253,278,284**) (including at line 236) to ensure accuracy and avoid confusion. The manuscript has been updated accordingly (Lines **253-254, 281, 284)**.

**Reviewer’s Comments to the Author (5)**

**Reviewer #1: Thank you for the opportunity to review your manuscript. This is an original and well-structured study reporting the molecular detection and genetic diversity of *Theileria orientalis* in cattle across Bangladesh. The large sample size, national coverage, and sequence confirmation make this work a valuable contribution to regional epidemiology and tick-borne disease surveillance. Overall, the study is technically sound, conclusions are supported by the data, and the English is clear. Only minor revisions are needed to enhance clarity and reporting consistency.**

**1. Sampling description**

**The random sampling strategy is appropriate and ensures diversity of age, breed, and sex. To enhance transparency, please briefly describe how randomization was implemented within farms, herds, or markets (e.g., random selection on site).**

**Response:**

Thank you for the constructive comment. We agree that providing additional details improves transparency. We have now clarified the implementation of randomization in the Methodology section. Specifically, we described that within each farm/herd, animals were selected using a simple random approach. The manuscript has been revised to include this information in the Methodology section (**Lines 98-99)**.

**2. PCR methodology**

**It is clear that positives were confirmed by sequencing, which supports assay specificity. Please indicate explicitly whether no-template or extraction controls were included.
Although homologous MPSP-like genes exist in *T. parva* and *T. annulata*, the primer pair used here targets a region unique to the *T. orientalis* complex (as validated in Kamau et al., 2011). Mentioning this would clarify assay specificity given the absence of local DNA from other *Theileria* species in Bangladesh.**

**Response:**

Thank you for the insightful comments. Specifically, we confirm that no-template (negative) control was included in all PCR runs. Additionally, we have added a statement regarding primer specificity. The MPSP primers target a region unique to the *T. orientalis* complex. Although *T. annulata* and *T. parva* possess homologous genes, this primer pair has been validated to amplify only *T. orientalis*, supporting the specificity of our assay in the absence of local DNA from other *Theileria* species. We have added this detail to the methodology section of the revised manuscript (**Lines 119-123**).

**3. Statistical reporting**

**The use of Pearson’s chi-square and Fisher’s exact tests (SPSS v27) is appropriate for the descriptive objective, and this information is already clearly stated in the Methods. Reporting 95% confidence intervals for prevalence estimates would enhance interpretability, but no further analysis is required.**

**Response:**

Thank you for this constructive suggestion. In accordance with the reviewer’s recommendation, 95% confidence intervals for all prevalence estimates have been calculated and incorporated into the relevant table (Table 1) in the revised manuscript. We believe this addition improves the clarity and interpretability of the descriptive epidemiological results of this study.

**4. Sequencing subset**

**A total of 510 bovines' blood was found positive with *T. orientalis*. Could you please state briefly how the 32 sequenced isolates were selected (randomly or by gel quality/representation). Clarify that genotype diversity reflects this subset and may not capture all variants nationwide.**

**Response:**

We appreciate the reviewer’s comment. The 32 sequenced isolates were chosen based on amplicons quality and geographic representation. This has been clarified in the revised manuscript. We also note that the detected genotype diversity reflects this subset and may not encompass all variants present nationwide. In response to the reviewer’s comment, we have added the relevant statement to the discussion section (**Lines 350-355**).

**5. Genotype classification**

**The study identifies *T. orientalis* MPSP genotypes 5 and 7, both of which have been reported previously in Asia. While these genotypes are not novel, documenting their concurrent circulation across multiple Bangladeshi divisions adds valuable epidemiological context. The authors could emphasize this contribution more clearly in the Discussion.**

**Response:**

We appreciate the reviewer’s thoughtful suggestion. Accordingly, the discussion section has been expanded for better understanding and highlighting the importance of identifying MPSP genotypes 5 and 7 circulating simultaneously across several divisions of Bangladesh. While these genotypes have been previously reported in Asia, their widespread presence in Bangladesh that adds meaningful epidemiological context and contributes to understanding regional transmission dynamics. This point has been duly addressed and incorporated in the discussion section **(Lines 310–316)**.

**6. Figures and presentation**

**Figure 1 (Map): Please cite the map source/shape file and ensure boundary lines are clearly visible in print.**

**Response**

We appreciate the reviewer’s suggestion. In the revised manuscript, we have replaced the previous image with a newly created map developed entirely by the authors based on the study area's administrative boundaries. Because this map was generated independently and does not use external shape file data, no citation is needed. The boundary lines have also been improved to ensure clarity in printing form.

**Figure 3 (Haplotype network): Please just verify label legibility (≥ 8 pt).**

**Response:**

We thank the reviewer for the suggestion. The labels in Figure 3 (Haplotype network) have been checked and adjusted to ensure legibility at ≥ 8 pt, as requested.

**7. Discussion**

**The Discussion is well written and adequately contextualizes the results within regional and global *T. orientalis* epidemiology. Comparative analysis with previous studies is informative. However, the section could be strengthened by briefly explaining the ecological or management factors influencing regional differences, and by adding a concise concluding paragraph linking the findings to surveillance or control strategies. Overall, this is a solid and publishable discussion after minor revisions.**

**The manuscript is written in Standard English. Minor typographical and taxonomic corrections (e.g., *Amblyomma testudinarium*, italicized species names, consistent “Chattogram”) can be made at proof stage.**

**Response:**

We appreciate the reviewer’s observation. All minor typographical and taxonomic issues have been addressed in the revised manuscript. This includes ensuring all species names are italicized and standardizing the use of “Chattogram” throughout the text. It is noted that, the Chittagong Division has been officially renamed as the **Chattogram Division**. The name change for the division and city from "Chittagong" to "Chattogram" **(Line 95, 158, 188, 191, 192, 196, 210, 212, 238, 268, 270, 290, 292)** (and several other districts) was made official in 2018 to better reflect their Bangla pronunciation. The official link for the Chattogram Division portal is: https://www.chittagongdiv.gov.bd/en

The specific spelling of *Amblyomma testudinarium* is no longer relevant because the entire sentence has been revised.
